# Response to: Comment on “Pain Perception of the First Eye versus the Second Eye during Phacoemulsification under Local Anesthesia for Patients Going through Cataract Surgery: A Systematic Review and Meta-Analysis”

**DOI:** 10.1155/2021/6834852

**Published:** 2021-01-06

**Authors:** Chuying Shi, Benny Zee

**Affiliations:** Division of Biostatistics, Jockey Club School of Public Health and Primary Care, Faculty of Medicine, The Chinese University of Hong Kong, New Territories, Hong Kong

We appreciate the interest of Sun et al. [1] in our article [2]. They suggested in their Letter to the Editor that we could provide detailed information on sedatives and analgesics administered to patients and perform subgroup analysis based on these confounding factors. There were eight studies included in our meta-analysis. Sharma et al. administered small doses of intravenous agents (midazolam, propofol, and fentanyl) to all individual patients who needed to achieve adequate relaxation on the day after the surgery [3]. Hari-Kovacs et al. routinely used 10 mg/os temazepam on the day ward [4]. Zhang et al. administered pranoprofen eye drops to patients in the treatment group only before their second-eye surgery, but did not mention any sedatives/analgesics in the control group [5]. One study did not describe the use of sedatives/analgesics in detail, and sedatives/analgesics were not routinely administered to all eyes in first-eye and second-eye surgery [6]. The remaining four of the eight studies used neither sedatives nor analgesics. In total, Sharma et al. and Hari-Kovacs et al. administered sedatives or analgesics in both first-eye and second-eye surgery. However, subgroup analysis cannot be performed because these two studies assessed pain on different days—one was on the day of surgery and another was on the day after surgery. With high levels of heterogeneity, meta-analyses would have low predictive values no matter what the *I*^2^ values are [7]. Therefore, we only did a subgroup analysis on the four studies that did not use any sedatives/analgesics. The result (WMD: 0.62; 95% CI: 0.53, 0.71; *P* < 0.00001; *I*^2^ = 0%) showed that the pain scores of the first eye on the day of the surgery were significantly lower compared to those of the second eye when no sedatives/analgesics were used, which was consistent with our original result. A possible pharmacological explanation of greater eye pain in second-eye surgery is that the previous exposure to analgesic and sedative medications during the first-eye surgery may cause drug tolerance so that the response to the same medications was decreased during the second-eye surgery. However, this seems unlikely according to the result of this subgroup analysis where no sedatives/analgesics were used. The meta-analysis of using sedatives/analgesics therefore requires more studies to further investigate.

Use of sedation and analgesia in patients undergoing ophthalmic surgery varies significantly both between and within countries. This is due to the wide variety of cultural expectations, cost, traditions, institutional practices, and availability of personnel and facilities [8]. The eight studies in our meta-analysis demonstrate this variability. The eight studies ranged from 2008 to 2018, and the countries involved included Australia, USA, UK, Turkey, and China. This is in addition to the different types of cataract surgeries and anesthesia methods utilized across the studies.

In conclusion, we thank Sun et al. for their interest in our study and for offering their valuable opinion and perspective from an anesthetist's angle. We hope this work helps stimulate both ophthalmologists and anesthetists to explore and re-evaluate how to manage pain in second-eye cataract surgery.
